# Enterococcal periprosthetic joint infection: clinical and microbiological findings from an 8-year retrospective cohort study

**DOI:** 10.1186/s12879-019-4691-y

**Published:** 2019-12-27

**Authors:** Nora Renz, Rihard Trebse, Doruk Akgün, Carsten Perka, Andrej Trampuz

**Affiliations:** 1 Center for Musculoskeletal Surgery (CMSC), Charité – Universitätsmedizin Berlin, corporate member of Freie Universität Berlin, Humboldt-Universität zu Berlin, and Berlin Institute of Health, Charitéplatz 1, 10117 Berlin, Germany; 2 Orthopaedic Hospital Valdoltra, SI-6280 Ankaran, Slovenia

**Keywords:** Periprosthetic joint infection, *Enterococcus* spp., Outcome, Microbiology, Biofilm

## Abstract

**Background:**

Treatment of enterococcal periprosthetic joint infections (PJI) is challenging due to non-standardized management strategies and lack of biofilm-active antibiotics. The optimal surgical and antimicrobial therapy are unknown. Therefore, we evaluated characteristics and outcome of enterococcal PJI.

**Methods:**

Consecutive patients with enterococcal PJI from two specialized orthopedic institutions were retrospectively analyzed. Both institutions are following the same diagnostic and treatment concepts. The probability of relapse-free survival was estimated using Kaplan-Meier survival curves and compared by log-rank test. Treatment success was defined by absence of relapse or persistence of PJI due to enterococci or death related to enterococcal PJI. Clinical success was defined by the infection-free status, no subsequent surgical intervention for persistent or perioperative infection after re-implantation and no PJI-related death within 3 months.

**Results:**

Included were 75 enterococcal PJI episodes, involving 41 hip, 30 knee, 2 elbow and 2 shoulder prostheses. PJI occurred postoperatively in 61 episodes (81%), hematogenously in 13 (17%) and by contiguous spread in one. *E. faecalis* grew in 64 episodes, *E. faecium* in 10 and *E. casseliflavus* in one episode(s). Additional microorganism(s) were isolated in 38 patients (51%). Enterococci were susceptible to vancomycin in 73 of 75 isolates (97%), to daptomycin in all 75 isolates, and to fosfomycin in 21 of 22 isolates (96%). The outcome data was available for 66 patients (88%). The treatment success after 3 years was 83.7% (95% confidence interval [CI]; 76.1–96.7%) and the clinical success was 67.5% (95% CI; 57.3–80.8%). In 11 patients (17%), a new PJI episode caused by a different pathogen occurred. All failures occurred within 3 years after surgery.

**Conclusion:**

About half of enterococcal PJI were polymicrobial infections. The treatment success was high (84%). All treatment failures occurred within the first 3 years after revision surgery. Interestingly, 17% of patients experienced a new PJI caused by another pathogen at a later stage.

**Trial registration:**

The study was retrospectively registered with the public clinical trial identification NCT0253022 at https://www.clinicaltrials.gov on 15 July 2015.

## Background

Enterococci are reported as causing pathogen of periprosthetic joint infection (PJI) in 2.3 to 15% [1, 2], often as part of a polymicrobial infection [3, 4]. Despite low virulence of the pathogen, the treatment of enterococcal PJI is challenging due to slow bactericidal activity of antimicrobial agents, antimicrobial tolerance and increasing resistance [5]. Data on biofilm-active antibiotics against enterococci are limited or not existing, hence enterococcal PJI were previously classified as “difficult-to-treat” [6]. In addition, some authors report high treatment failure rates [7–9].

Management strategies in enterococcal PJI are controversial. Depending on the surgical procedure applied, divergent outcome results are reported [1, 10–12]. Similarly, antimicrobial treatment recommendations vary widely and predominantly originate from in vitro and experimental studies or are extrapolated from the guidelines for treatment of infective endocarditis. Most authors recommend aminopenicillin derivatives in penicillin-susceptible isolates, whereas vancomycin and linezolid are suggested for penicillin-resistant enterococci. Some experts propose single antimicrobial therapy, while others recommend combination treatment with aminoglycosides or ceftriaxone [6, 13–15]. Furthermore, the role of rifampin is controversial [16, 17]. Newer antibiotic options such as daptomycin, lipoglycopeptides and fosfomycin are even less investigated for enterococcal PJI [17–19].

We performed a retrospective cohort study in two orthopedic clinics specialized in endoprothetic septic surgery, which are following the same diagnostic and treatment concepts. The aim of the study was to analyze the clinical, laboratory and microbiological characteristics, treatment modalities and outcome of enterococcal PJI.

## Methods

### Study design and population

This retrospective cohort study was conducted in two large orthopedic centers, having a specialized septic surgery unit. PJI episodes caused by *Enterococcus* species, alone or in combination with another microorganism(s), treated at one of the participating institutions from January 2010 to December 2017 were included. Data on PJI were collected using the same definition criteria, diagnostic procedure and outcome evaluation. The study was approved by the lead ethical committee (approval No. EA04/040/14) and was conducted in accordance with the Declaration of Helsinki. The study was registered with the public clinical trial identification NCT0253022 at https://www.clinicaltrials.gov.

### Study definitions

PJI was defined according to the following definition criteria, as previously used [20–22]. According to these criteria, at least one of the following criteria are needed for the diagnosis of PJI: (i) macroscopic purulence surrounding the prosthesis, (ii) presence of communicating sinus tract, (iii) increased absolute synovial fluid leukocyte count or differential (> 2000 leukocytes/μl or > 70% granulocytes), (iv) isolation of enterococci from synovial fluid, periprosthetic tissue or sonication culture, (v) positive histopathology, defined as > 23 granulocytes per 10 high-power fields, corresponding to type II or type III periprosthetic membrane [23]. If enterococci grew in only one microbiological specimen, the microbiological finding was sufficient for the diagnosis of PJI only if additional (non-microbiological) criterion was present, as defined above.

Infections were classified according to their temporal appearance after surgery into early (< 3 months), delayed (3–24 months) and late infection (> 24 months) [24]. In addition, infections were defined as acute PJI (new onset symptoms for ≤4 weeks), or chronic PJI (symptoms duration > 4 weeks). The hematogenous route of PJI was defined when (i) the onset of the symptoms was > 3 months after implantation and occurred after an initial uneventful initial course and (ii) the infection presented with acute onset or the same *Enterococcus* species grew in blood cultures or from a distant infectious focus. Each case was evaluated and classified by an interdisciplinary team of orthopedic surgeons (DA, RT, CP) and infectious disease specialists (AT, NR).

*Infection-free interval* describes the interval from primary implantation or last septic surgery of the prosthesis to the diagnosis of an enterococcal infection.

*Treatment success* was defined by absence of relapse or persistence of PJI due to enterococci or death related to enterococcal PJI.

*Clinical success* was defined by the presence of all following criteria at last follow-up: (i) infection-free status, characterized by a healed wound without fistula, drainage, and no recurrence of the infection, (ii) no subsequent surgical intervention for persistent or perioperative infection, and (iii) no PJI-related death (within 3 months).

### Microbiological testing

An automated broth microdilution assay was used to determine the antimicrobial susceptibility of all antibiotics except for fosfomycin, and the results were interpreted according to European Committee on Antimicrobial Susceptibility Testing (EUCAST) criteria. For fosfomycin, Etest (bioMérieux, Marcy-l’Étoile, France) was performed in Müller Hinton agar (BD, Heidelberg, Germany) according to manufacturer’s instructions. After incubation at 37 °C for 24 h, the minimal inhibitory concentration (MIC) was recorded as the concentration value where the inhibition ellipse intersected the scale of the strip.

### Surgical treatment

All patients underwent revision surgery. Patients with acute (early postoperative or late hematogenous) infection with symptoms lasting < 4 weeks were treated with retention of the prosthesis, change of the mobile parts and meticulous debridement. In contrast, patients with chronic PJI, with signs of infection lasting ≥4 weeks or with a loose prosthesis were treated with one-stage or two-stage revision, depending on the local soft tissue and bone conditions and the revision history.

### Antimicrobial treatment

Empiric antibiotic treatment was started intravenously after surgery and was subsequently adapted according to the susceptibility of the isolated organism. The intravenous treatment was typically continued for at least 2 weeks, followed by oral antibiotic treatment, as previously described [25, 26]. In case of a two-stage revision, antibiotics were administered without interruption until re-implantation. After re-implantation, antibiotics were continued to complete a total duration of at least 12 weeks (or longer, if the prosthesis-free interval was > 6 weeks).

Adequate antimicrobial therapy was considered when the antibiotic was appropriate against enterococcal infection according to its activity, dose, oral bioavailability and bone penetration. The antibiotics were chosen according to institutional treatment guidelines and patient history of antibiotic allergies. The following intravenous doses were used in patients with normal renal function: vancomycin 15–20 mg/kg every 12 h, daptomycin 8–10 mg/kg once daily, fosfomycin 5 g every 8 h, penicillin G 5 million IU every 6 h, ampicillin 2 g every 6 h, gentamicin 3 mg/kg once daily. In case of concomitant infective endocarditis, higher doses were administered according to the respective guidelines.

### Follow-up evaluation

Patients were scheduled for follow-up in the outpatient clinic at 3, 6, 12 and 24 months after revision surgery. Clinical, laboratory and radiological evaluation was performed and interpreted interdisciplinary by an orthopedic surgeon and an infectious disease specialist. Further follow-up evaluation was performed by phone contact using a standardized case-report form.

### Statistical analysis

Categorical variables were compared using the Fisher’s exact test, for comparison of continuous variables the Mann-Whitney U test was applied. A two-sided *p*-value of < 0.05 was considered significant. The probability of infection-free survival and 95% confidence interval (CI) was estimated using the Kaplan-Meier survival method. Outcomes between groups were compared using Fishers exact test. An alpha level of 0.05 was considered significant. For statistical analysis and graphics, Prism software (version 8.2; GraphPad, La Jolla, CA, USA) was used.

## Results

### Patient demographical data

A total of 75 patients were included. The demographics of 37 monomicrobial and 38 polymicrobial enterococcal PJI are shown in Table 1. At least one previous revision surgery was performed in 61 patients (81%), the number of previous surgeries ranged from 1 to 18 interventions.

**Table 1:** Patient demographics and infection characteristics of 75 patient with enterococcal PJI

Variable	All episodes (*n* = 75)	Monomicrobial (*n* = 37)	Polymicrobial (*n* = 38)	*P* value
Female sex, No. (%)	48 (64)	21 (57)	27 (71)	0.234
Median age (range) - years	76 (30–90)	78 (52–90)	75 (30–89)	0.810
Affected joint, No. (%)				
Hip	41 (55)	21 (57)	20 (53)	0.818
Knee	30 (40)	12 (32)	18 (47)	0.241
Elbow	2 (3)	2 (5)	–	0.240
Shoulder	2 (3)	2 (5)	–	0.240
Previous revision surgery, No. (%)	61 (81)	27 (73)	34 (89)	0.082
Septic revision	34 (56)	16 (59)	18 (53)	
Aseptic revision	27 (44)	11 (41)	16 (47)	

### Infection characteristics (Table 2)

Twenty-seven episodes (36%) occurred early after surgery, 30 (40%) delayed and 18 (24%) late.

**Table 2:** Infection characteristics of 75 patient with enterococcal PJI

Variable	All episodes (*n* = 75)	Monomicrobial (*n* = 37)	Polymicrobial (*n* = 38)	*P* value
Infection manifestation according to the time after last surgery				
Early	27 (36)	10 (27)	17 (45)	0.150
Delayed	30 (40)	17 (46)	13 (34)	0.351
Late	18 (24)	10 (27)	8 (21)	0.597
Median delay from last revision to PJI (range) - months	8.7 (0.5–336)	10.2 (0.3–336)	5.0 (0.4–133)	0.588
Median delay from primary implantation of prosthesis to PJI (range) - months	47.7 (0.3–418)	36.4 (0.3–418)	56.6 (0.4–373)	0.810
Presumed route of infection				
Perioperative^a^	61 (81)	25 (68)	36 (95)	0.003
Hematogenous	13 (17)	12 (32)	1 (3)	< 0.001
Direct extension by adjacent infectious focus (contiguous)	1 (1)	–	1 (3)	
Signs and symptoms				
Joint pain	48/70 (69)	22/32 (69)	26/38 (68)	1.000
Local signs	46 (61)	20 (54)	26 (68)	0.241
Sinus tract	16 (21)	3 (8)	13 (34)	0.010
Prosthesis loosening in x-ray	16 (21)	5 (14)	11 (29)	0.158
Laboratory results at admission				
Serum C-reactive protein				
Median (range) - mg/l	36 (1–295)	45 (12–229)	36 (1–295)	0.936
Increased (> 10 mg/l)	63 (84)	30 (81)	33 (87)	0.544
White blood cell count				
Median (range) – G/l	7.9 (3.2–34.23)	7.4 (4.8–22.4)	8.3 (3.2–34.3)	0.992
Increased (> 10 G/l)	18 (24)	8 (22)	10 (26)	0.788
Synovial fluid leukocyte count				
Increased value^b^	25/29 (86)	16/17 (94)	9/12 (75)	0.279
Median absolute count (range) - 10^3^/μl	20.3 (0.42–160)	23 (1.8–160)	7.1 (0.4–140)	0.097
Histopathology consistent with infection	53/61 (87)	26/28 (93)	27/33 (82)	0.294

Data are no. (%) of patients, unless otherwise indicated. Where the denominator is shown, data was not available for all patients

^a^Among them 13 cases (9 polymicrobial and 4 monomicrobial) occurred during surgical and antimicrobial treatment of a PJI caused by another pathogen

^b^Increased absolute number of leukocytes or percentage of granulocytes

The presumed route of infection was perioperative in the majority of PJI (81%), whereas the hematogenous route was documented in 13 episodes (17%). The primary infection focus was found in 7 cases, including infective endocarditis in four patients and urinary tract infection in three patients. In 13 patients, enterococci were detected as second pathogen, isolated during treatment of PJI initially caused by another pathogen.

Enterococcal PJI presented with acute onset in 33 patients (44%) and with chronic symptoms in 42 (56%). Sinus tract was more often observed in polymicrobial than monomicrobial infections (34% vs. 8%, *p* = 0.010).

Among diagnostic tests, positivity rate of histopathology of periprosthetic tissue (87%), synovial fluid leukocyte count (86%) and serum C-reactive protein concentration (84%) were high, whereas only 18 patients (24%) presented with elevated serum white blood cell count.

### Microbiological findings

Table 3 summarizes the isolated enterococcal species and source of pathogen isolation. PJI was caused by *E. faecalis* in 64 patients (85%), by *E. faecium* in 10 patients (13%) and by E. casseliflavus in 1 patient (1%). The most common co-pathogens in polymicrobial infections were coagulase-negative staphylococci and gram-negative bacilli.

**Table 3:** Microbiology of 75 patient with enterococcal PJI

Variable	All episodes (*n* = 75)	Monomicrobial (*n* = 37)	Polymicrobial (*n* = 38)	*P* value
*Enterococcus* spp.				
*E. faecalis*	64 (85)	33 (89)	31 (82)	0.516
*E. faecium*^a^	10 (13)	4 (11)	6 (16)	0.736
*E. casseliflavus*	1 (1)	-	1 (3)	1.000
Co-pathogens in mixed infections				
Coagulase-negative staphylococci^b^			20	
Gram-negative bacilli^c^			18	
Anaerobes^d^			5	
*Candida* spp^e^			4	
*Streptococcus* spp^f^			3	
*Staphylococcus aureus*			2	
*Corynebacterium* spp^g^			2	
Source of pathogen isolation				
Synovial fluid	42/55 (76)	20/25 (80)	22/30 (73)	0.752
Periprosthetic tissue	52/67 (78)	24/33 (73)	28/34 (82)	0.392
Sonication fluid	33/45 (73)	15/22 (68)	18/23 (78)	0.514

Data are no. (%) of patients, unless otherwise indicated. Where the denominator is shown, data was not available for all patients

^a^Including 2 vancomycin-resistant enterococci (VRE)

^b^*S. epidermidis* (*n* = 14), *S. capitis* (*n* = 2), *S. lugdunensis* (*n* = 2), *S. saccharolyticus* (*n* = 1) and *S. haemolyticus* (*n* = 1)

^c^*Klebsiella* spp. (*n* = 6), *E. coli* (*n* = 5), *Proteus* spp. (*n* = 5), *Pseudomonas aeruginosa* (*n* = 4), *Morganella* spp. (*n* = 2), *Serratia marcescens* (*n* = 1), *Enterobacter cloacae* (*n* = 1), A*cinetobacter* spp. (*n* = 1)

^d^*Finegoldia magna* (*n* = 3), *Peptinophilus asaccharolyticus* (*n* = 1), *Peptostreptococcus micros* (*n* = 1), *Bacteroides fragilis* (*n* = 1)

^e^*C. albicans* (*n* = 4), *C. parapsilosis* (*n* = 1)

^f^*S. salivarius* (*n* = 1), *S. cristatus* (*n* = 1), not further specified (*n* = 1)

^g^*C. striatum* (*n* = 1), *C. tuberculostearicum* (*n* = 1)

**Fig. 1:**
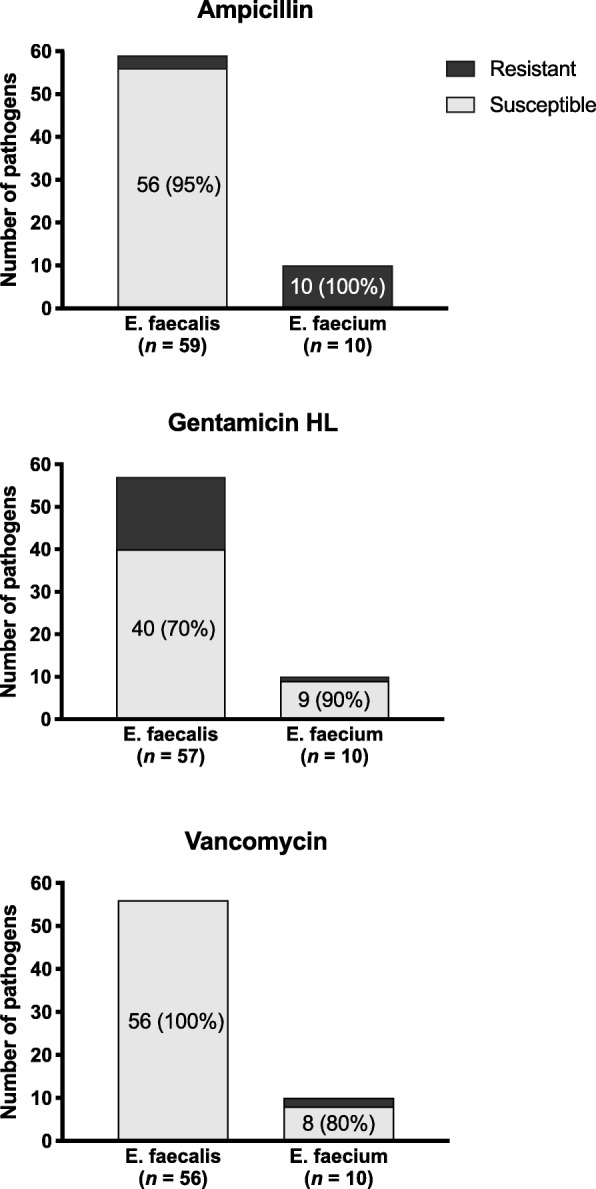
Susceptibility of enterococcus isolates to ampicillin, gentamicin high-level and vancomycin. HL, high-level

**Fig. 2:**
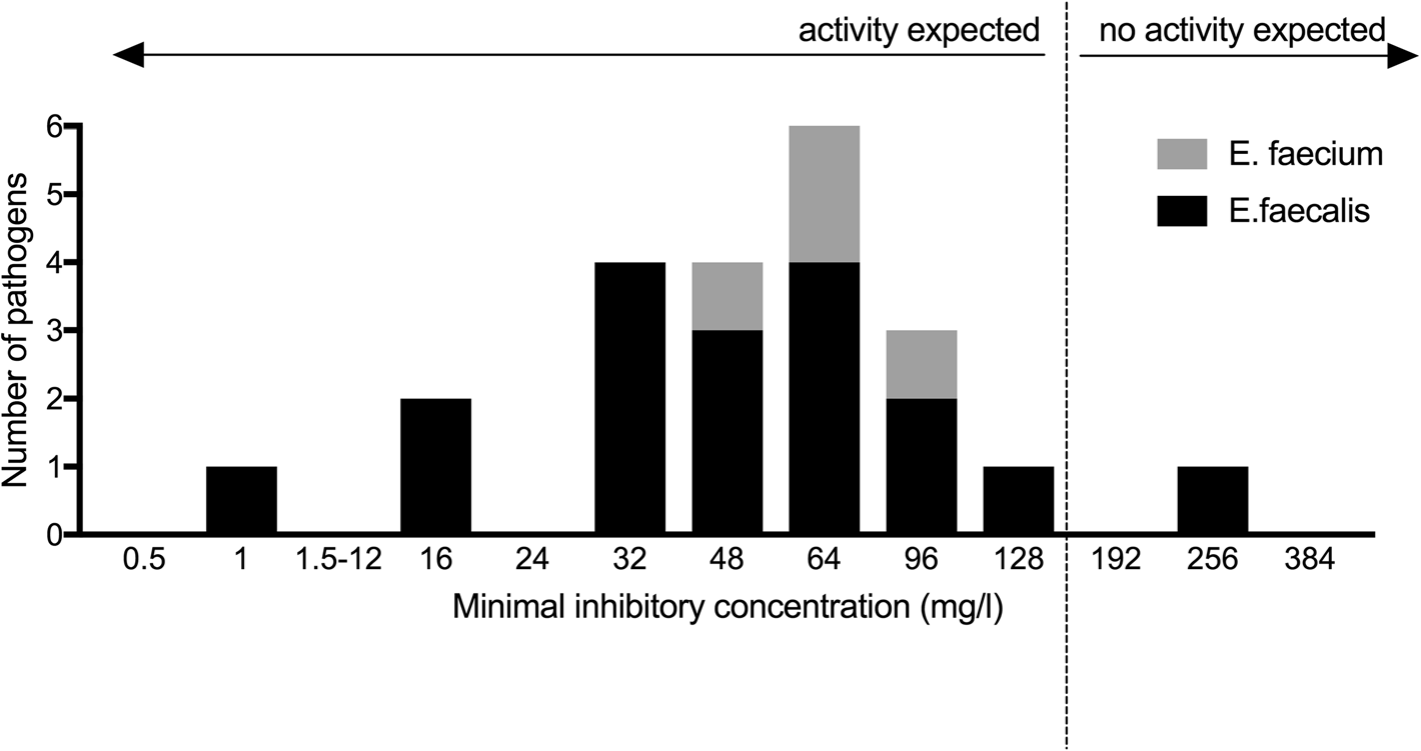
Minimal inhibitory concentrations of enterococcal isolates for fosfomycin (*n* = 22)

Figure 1 summarizes the antimicrobial susceptibilities of enterococci to ampicillin, high-level of gentamicin and vancomycin. Of 22 enterococcal isolates, 21 had an MIC for fosfomycin of ≤128 mg/l (Fig. 2).

In 13 patients, only one specimen (tissue in 4 patients and synovial fluid in 9 patients) grew the *Enterococcus* spp. In all of them, a non-microbiological criterion was fulfilled, i.e. among positive histopathology in 9 patients, purulent secretion in 3 patients and positive synovial fluid leukocyte count in 1 patient.

### Surgical treatment

Table 4 summarizes the surgical and antimicrobial treatment of analyzed patients. Retention of the prosthesis was performed in 13 patients with acute infection, one-stage exchange in 10 patients (2 acute and 8 chronic PJI) and two-stage exchange in 43 patients. The median interval between explantation and re-implantation surgery was 84 days (range, 29–292 days). In 9 patients (4 hips, 4 knees, 1 elbow) resection arthroplasty was performed.

**Table 4:** Surgical and antimicrobial treatment of 75 patient with enterococcal PJI

	All episodes (*n* = 75)	Mono-microbial (*n* = 37)	Poly-microbial (*n* = 38)	*P* value
Surgical treatment				
Prosthesis retention^a^	13 (17)	7	6	0.768
One-stage exchange^b^	10 (13)	5	5	1.000
Multi-stage exchange^c^	43 (57)	20	23	0.644
No. surgical interventions needed, median (range)	2 (2–8)	2 (2–5)	3 (2–8)	0.039
Resection arthroplasty	9 (12)	5	4	0.736
Antimicrobial treatment^d^				
Intravenous antibiotic agent				
Penicillin derivative	61/74 (82)	30/37 (81)	31/37 (84)	1.000
Vancomycin or daptomycin	12/74 (16)	6/37 (16)	6/37 (16)	1.000
Other	1/74 (3)	1/37 (3)	–	
Additive agent for combination treatment				
Fosfomycin	17/74 (23)	9/37 (24)	8/37 (22)	1.000
Gentamicin	16/74 (22)	11/37 (30)	5/37 (14)	0.157
Fosfomycin and gentamicin	8/74 (11)	5/37 (14)	3/37 (8)	0.711
Vancomycin or daptomycin	18/74 (24)	4/37 (11)	14/37 (38)	0.013
None	15/74 (20)	8/37 (22)	7/37 (20)	1.000
Median total duration (range) – weeks^d^	16 (2–52)	15 (4–39)	16 (2–52)	0.459
Prolonged treatment duration (> 12 weeks)	35/74 (47)	17/37 (46)	18/37 (49)	1.000

Data are no. (%) of patients, unless otherwise indicated. Where the denominator is shown, data was not available for all patients

^a^Including exchange of mobile parts (*n* = 7), debridement only (*n* = 4) and no surgery (*n* = 1)

^b^In 3 patients, only partial exchange was performed

^c^Median interval between explantation and re-implantation was 84 days (range, 29–292 days)

^d^One patient received no antimicrobial treatment

### Antimicrobial treatment

Antimicrobial therapy was administered in 74 patients (99%), all of them were initially treated with intravenous antibiotics. Fifty-nine patients (80%) received a combination therapy with the addition of gentamicin (*n* = 24), vancomycin or daptomycin (*n* = 18) or fosfomycin (*n* = 25). The median duration of fosfomycin treatment was 14 days (range, 3–90 days).

The antibiotic treatment was considered adequate in 66 patients (88%), which included either penicillin derivative (in ampicillin-susceptible enterococci) or vancomycin or daptomycin (in ampicillin-resistant enterococci). The median duration of antibiotic treatment was 16 weeks (range, 2–52 weeks).

### Outcome evaluation

**Fig. 3:**
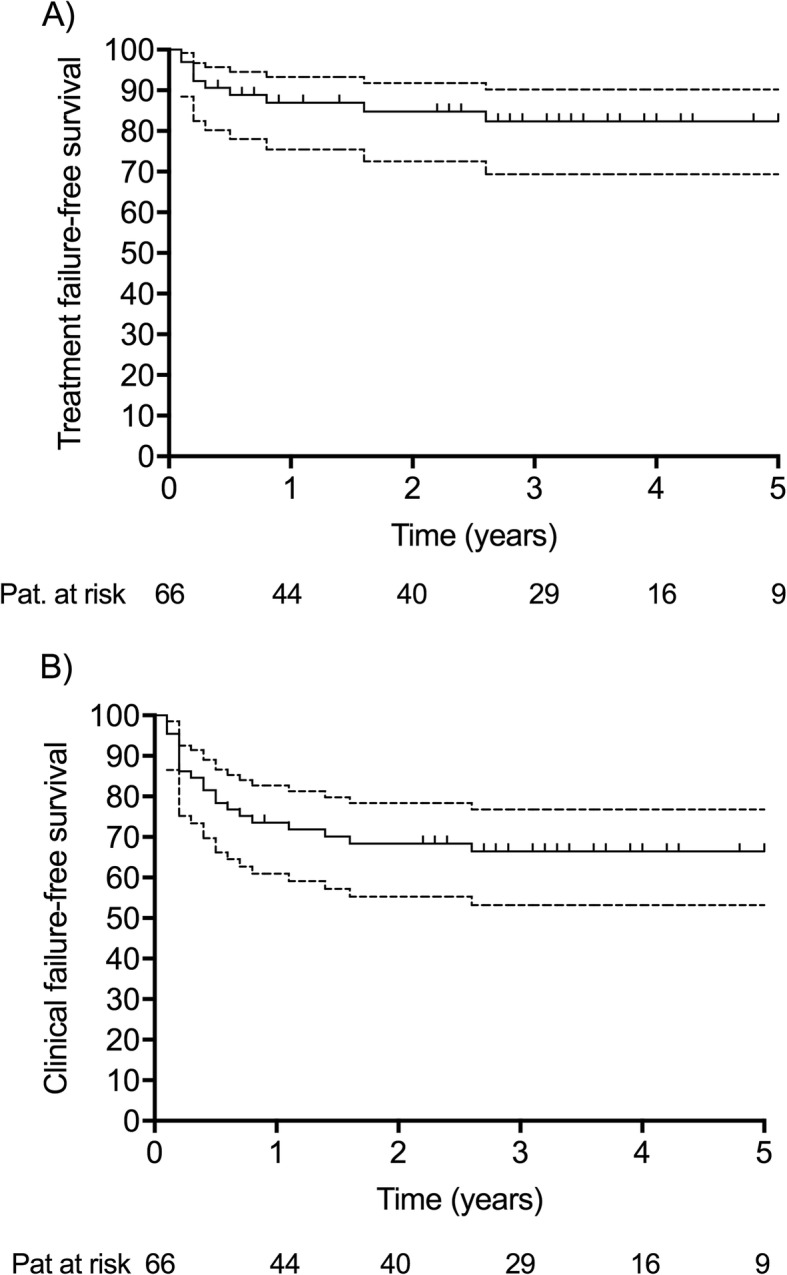
Outcome analysis of 75 patient with enterococcal PJI. Treatment failure (**a**) and clinical failure (**b**). Dotted lines indicate the 95% confidence interval

Table 5 shows outcome data of 66 patients (88%) for whom the follow-up data was available and who completed antibiotic therapy. The median follow-up was 31.8 months (range, 0.3–83.3 months). The relapse-free probability (treatment success) after 3 years was 83.7 (95% CI; 76.1–96.7%) and the infection-free probability (clinical success) was 67.5% (95% CI; 57.3–80.8%) (Fig. 3). All failures occurred within 3 years after diagnosis of enterococcal PJI. Among 21 patients with clinical failure, 10 experienced persistent infection or relapse due to enterococci (treatment failure) and 11 experienced new infection with another microorganism or culture-negative (nine of which occurred within 12 months). Two patients died within the first 3 months after surgical revision related to infection. Of 21 patients with clinical failure, 18 (86%) had previous revision surgery.

**Table 5:** Clinical outcome of 75 patient with enterococcal PJI

Outcome	All episodes (*n* = 66)	Monomicrobial (*n* = 33)	Polymicrobial (*n* = 33)	*P* value
Median follow-up (range) – months	31.8 (0.3–83.8)	25.9 (1.4–78.6)	33.8 (0.3–83.8)	0.138
Clinical success	45 (68)	22 (66)	23 (70)	1.000
Clinical failure^a^	21 (31)	11 (33)	10 (30)	
Isolation of another or no pathogen	11	5	6	
Treatment failure^b^	10	6	4	
Treatment success	56 (85)	27 (82)	29 (88)	0.752

Data are no. (%) of patients, unless otherwise indicated. Where the denominator is shown, data was not available for all patients

^a^6 patients died, 2 death were associated with the enterococcal PJI (treatment failure), 1 death was related to a new infection caused by staphylococci in the later course (clinical failure) and 3 patients died from a non-infectious cause (1 tumor, 2 pulmonary embolism) and were considered infection-free at the time of death

^b^Persistent infection or relapse with same *Enterococcus* spp. as initially isolated

**Fig. 4:**
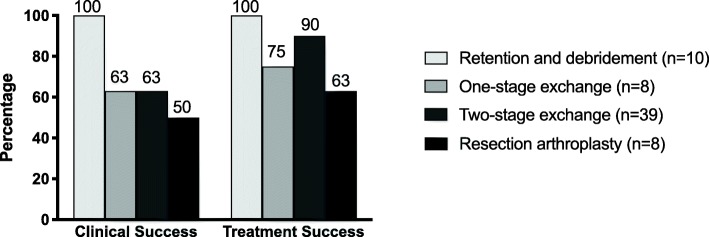
Clinical and treatment success depending on the performed surgical procedure in 66 patients with enterococcal PJI with available follow up. One patient in whom no surgery was performed experienced treatment failure

Clinical and treatment outcome according to surgical procedures is summarized in Fig. 4. Polymicrobial and monomicrobial infections showed no difference regarding treatment outcome (88% vs. 82%, *p* = 0.733). Adequate antimicrobial treatment was associated with better treatment outcome compared to inadequate therapy (91% vs. 38%, *p* = 0.002). No statistical difference was observed in the treatment outcome in patients receiving antimicrobial monotherapy or combination therapy (88% vs. 73%, *p* = 0.217), neither in those receiving fosfomycin in their treatment regimen (95%) compared to those without fosfomycin (80%) (*p* = 0.261). Prolonged antimicrobial therapy of > 12 weeks was also not associated with better treatment outcome than shorter therapy (81% vs. 85%, respectively, *p* = 0.920).

## Discussion

Outcome reports of enterococcal PJI vary widely, to a certain extent due to heterogeneous definition of treatment success. While some authors defined failure as relapse or persistent infection with isolation of the same pathogen, others also considered new infections caused by other pathogens as failures. In our study, failures were subclassified as treatment or clinical failure and success, respectively. Considering these definitions, the eradication of enterococcal PJI (treatment success) in our cohort was 83.7%, which was higher than previously reported (50 to 76%) [1, 9, 12, 27]. However, as 11 patients experienced new infection with another microorganism or were culture-negative, the infection-free probability (clinical success) was only 67.5%.

Rasouli et al. [1] described clinical success in 32 of 36 patients (89%) and treatment success in 35 of 36 patients (97%) with enterococcal PJI. However, in several patients additional major surgeries were performed such as resection arthroplasty, two-stage exchange, above-knee amputation or fusion. The fact that in our study no new PJIs occurred in patients with prosthesis retention and only in one patient undergoing one-stage exchange, corroborates the assumption that multiple surgeries may be a risk factor for introduction of a new pathogen. It remains unclear whether these infections are really “new” or a relapse of an unrecognized initial pathogen. Whereas adequate antimicrobial treatment was associated with better treatment outcome, prolonged antimicrobial therapy of > 12 weeks did not influence the outcome.

The EUCAST recommendations do not provide breakpoints for enterococci and fosfomycin and, thus, this substance is not recommended as monotherapy for the treatment of enterococcal infections. However, in vitro and animal model data showed activity of fosfomycin against planktonic and adherent *E. faecalis*. The most efficient regimen for killing planktonic and adherent *E. faecalis* was the combination of fosfomycin and gentamicin [17]. Extrapolated from the recommendation for the use of fosfomycin as combination partner for the treatment of infections caused by wild type isolates of *Pseudomonas* spp. with an epidemiological cut-off of 128 mg/l [28], the same cut-off is also used for enterococci and intravenous fosfomycin in daily routine. In our cohort, fosfomycin was active against most enterococcal isolates using the aforementioned fosfomycin MIC cut-off value. When intravenous fosfomycin (5 g every 8 h intravenously) was included in the treatment regimen, the treatment outcome was numerically higher than without intravenous fosfomycin (95% vs. 80%), however, the difference did not reach statistical significance. Prospective randomized controlled trials are needed to further explore the role of fosfomycin in biofilm-related infections.

Debridement and prosthesis retention was associated with the highest treatment success; however, this observation is most likely biased by rather treating “uncomplicated” and acute infections with retention and more complex ones with prosthesis removal. This fact represents the main limitation of the study. Other studies reported low clinical success with debridement and retention such as 40% [9], 47% [27], 50% [1] and 66% [11] and based on this observation two-stage prosthesis exchange is recommended for enterococcal PJI. However, with improved antimicrobial therapy, debridement and retention or one-stage prosthesis exchange may become an alternative to two-stage exchange with high treatment success. Based on our data, in our institution PJI caused by enterococci are no longer managed as difficult-to-treat infections as previously suggested [6]. Of note, permanent removal of prosthesis (resection arthroplasty without reimplantation) was performed in in 9 patients (12%) in our cohort. These patients were cured from infection, although the result may represent a functional inferior outcome, if they were able to walk before occurrence of PJI. Reasons for choosing resection arthroplasty were high risk of complications due to additional surgery, exceptionally insufficient bone stock or bedriddeness of patients.

In a previous study, the clinical presentation of enterococcal PJI was similar to that caused by low-virulent organisms such as *Cutibacterium acnes* and coagulase-negative staphylococci [12]. Accordingly, it remains unclear whether enterococci are true pathogens or contaminants when grown in a single specimen. If isolated in a single specimen, typical members of the normal skin flora are considered contaminants. As enterococci are not typically colonizing the skin, contamination during sampling appears unlikely. A similar question was investigated by Jindai et al. for positive blood cultures for enterococci [29]. The authors concluded that single-positive blood cultures likely indicate true infection rather than contamination, as the clinical outcome of patients with single-positive blood cultures was similar to those with multiple-positive blood cultures. In our study at least two positive *Enterococcus* spp. cultures were required, as defined by other authors evaluating enterococcal PJI [1, 9, 12]. However, a single positive culture was accepted if an additional non-microbiological criterion was present.

All treatment failures in our cohort occurred within the first 3 years after treatment of enterococcal PJI. Based on this finding, we recommend close follow-up in the first years, whereas the likelihood of relapse afterwards seems low. The risk of hematogenous PJI, however, remains during the whole indwelling time of the prosthesis, as recently demonstrated [30].

## Conclusions

Although the retrospective design of the study and the limited number of patients make definitive conclusions regarding outcome difficult, some important finding can be deduced from the presented study. First, about half of enterococcal PJI were polymicrobial infections. Second, the treatment success was unexpectedly high (84%), suggesting that enterococcal PJI are not difficult to treat. All treatment failures occurred within the first 3 years after revision surgery. Third, 17% of patients experienced a new PJI caused by another pathogen at a later stage. Fourth, intravenous fosfomycin was active against 21 of 22 enterococcal isolates (MIC ≤128 μg/ml) and its value should be further explored in prospective clinical trials involving enterococcal PJI.

## Data Availability

The datasets used and/or analysed during the current study are available from the corresponding author on reasonable request.

## Abbreviations

CI: Confidence interval
EUCAST: European Committee on Antimicrobial Susceptibility Testing
MIC: Minimal inhibitory concentration
PJI: Periprosthetic joint infections
